# Supplementary figures and images for: Various miRNAs compensate the role of miR-122 on HCV replication
Source: PLoS Pathog. 2020 Jun 23;16(6):e1008308. doi: 10.1371/journal.ppat.1008308 (PMC7337399; doi:10.1371/journal.ppat.1008308)

**GAGUG  
motif**

miR-122                    5'-UG**GAGUG**UGACAAUGGUGUUUG-3'  
miR-122-GAGUG        5'-UC**GAGUG****A**GACAAUGGUGUUUG-3'

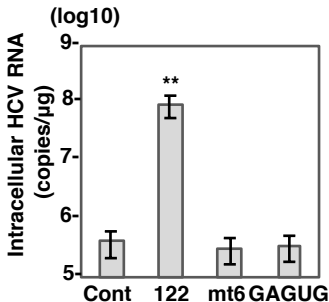

Supplement: S1 Fig — Sequence alignment of miR-122 and miR-122-GAGUG. Mismatching nucleotides were shown in red (top). Intracellular HCV-RNA levels of 751-122KO cells infected with JFH1 in the presence of mimic control, miR-122, miR-122-mt6 and miR-122-GAAGUG were determined at 72 hpi by qRT-PCR (bottom). (PDF) [file ppat.1008308.s001.pdf]

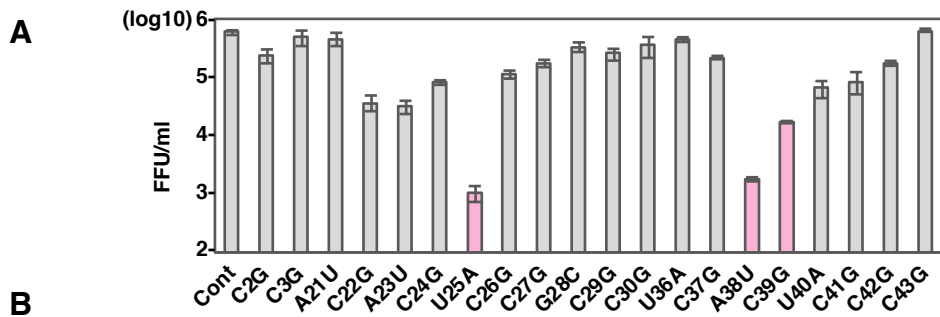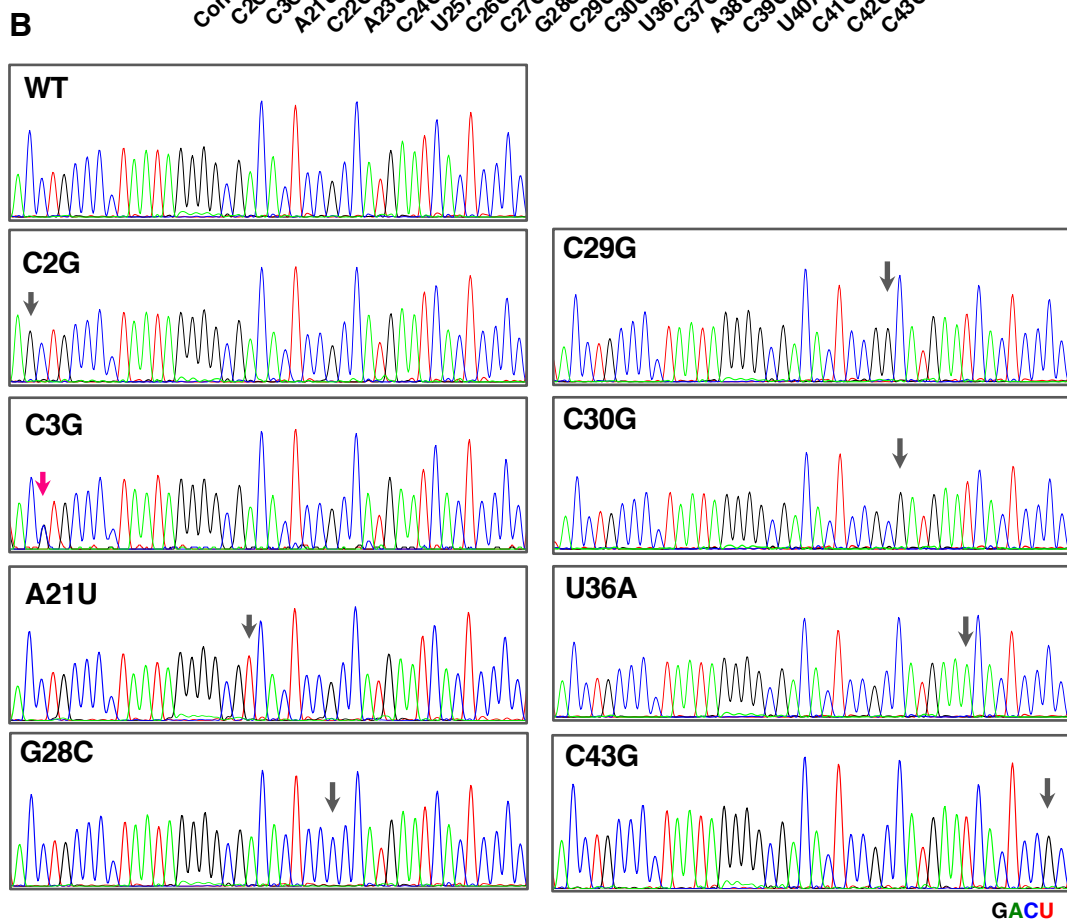

Supplement: S2 Fig — (A) The rescue of replication of HCV-RNA in 751-122KO cells by the expression of miR-122 variants. Various pHH-JFH1 plasmids encoding HCV mutants used in Fig 1D and corresponding miR-122 variants were transfected into 751-122KO cells and infectious titers in the culture supernatants at 3 dpi were determined by focus formation assay. Error bars indicate the standard deviation of the mean. (B) 5’RACE analysis of mutants with mutations in the 5’UTR of wild type, C2G, C3G, A21U, G28C, C29G, C30G, U36A and C43G. Sequences of 5’UTR of various HCV propagated in Huh7.5.1 cells were identified. Arrows indicate the position of each mutation in the 5’UTR of HCV and red arrow shows revertant. Each RNA base is represented as a colored peak: A, green; U, red; G, black; and C, blue. (PDF) [file ppat.1008308.s002.pdf]

**A**

| HCV-RNA genotype2a | TSI  |
|--------------------|------|
| miR-122-5p         | 0.97 |
| miR-504-3p         |      |
| miR-574-5p         | 0.79 |
| miR-3659           |      |
| miR-1236-5p        | 0.68 |
| miR-4481           | 0.68 |
| miR-4745-5p        | 0.71 |
| miR-4765           |      |
| miR-25-5p          |      |
| miR-4730           |      |
| miR-652-3p         | 0.62 |

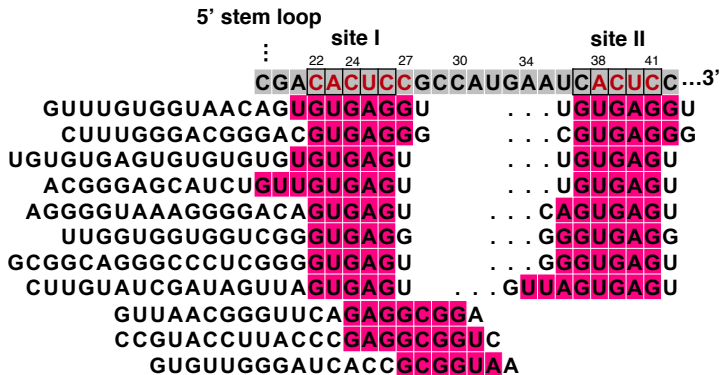**B**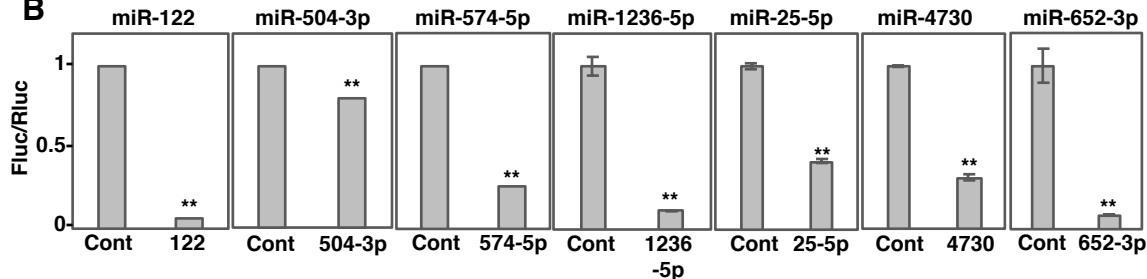

Supplement: S3 Fig — (A) Sequence alignment of interaction sites between HCV 5’UTR and miR- 122 type/non-miR-122 type miRNAs. Matching nucleotides were shown in red boxes. Nucleotides in site I and II of HCV-RNA were shown in red. (B) The activities of miR-122, miR-504-3p, miR-574-5p, miR-1236-5p (miR-122 type), miR-25-5p, miR4730 (non-miR-122 type) and miR-652-5p in miR-122- knockout Huh7.5.1 cells. pmirGLO vectors carrying the complementary sequence of each miRNA under the luciferase gene and each corresponding miRNA mimic were transfected into 751-122KO cells. At 24 h post-transfection, the luciferase activity was determined. The data are representative of three independent experiments. Error bars indicate the standard deviation of the mean and asterisks indicate significant differences (**P < 0.01) versus the results for the control. (PDF) [file ppat.1008308.s003.pdf]

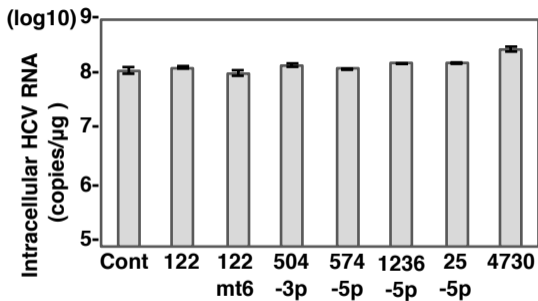

Supplement: S4 Fig — Intracellular HCV-RNA levels of Huh7.5.1 cells infected with JFH1 in the presence of mimic control, miR-122, miR-122-mt6, -504-3p, -574-5p, -1236-5p, -25-5p and -4730 were determined at 72 hpi by qRT-PCR. (PDF) [file ppat.1008308.s004.pdf]

**A**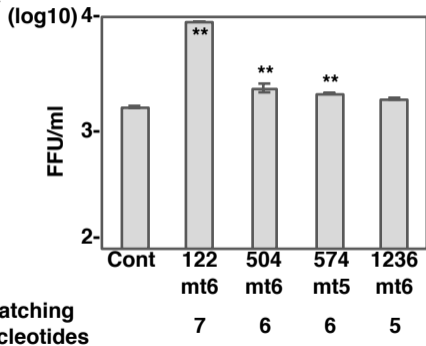**B**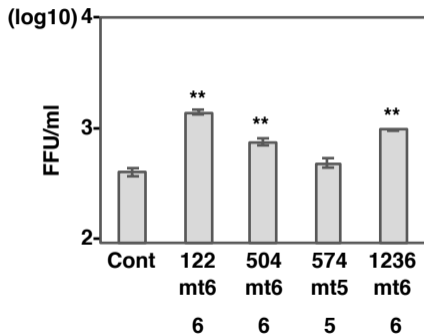

Supplement: S5 Fig — Intracellular HCV-RNA levels of 751-122KO cells transfected with pHH-JFH1mt-A23U (mutation in site I: A) and pHH-JFH1mt-A38U (mutation in site II: B) in the presence of mimic control, miR-122-mt6, -504-mt6, -574-mt5 and -1236-mt6 were determined at 72 hpi by qRT-PCR. Asterisks indicate significant differences (**P < 0.01). (PDF) [file ppat.1008308.s005.pdf]

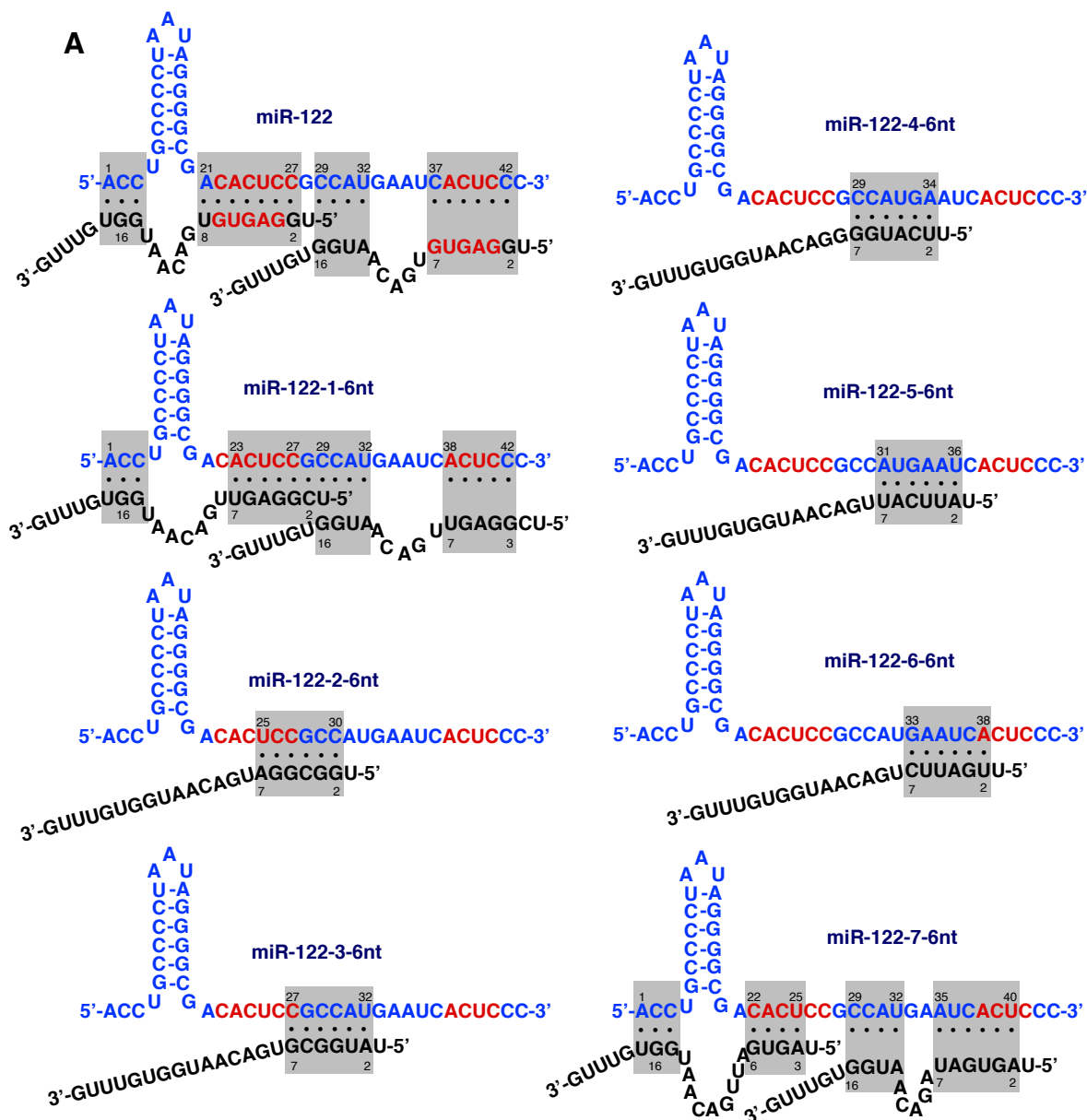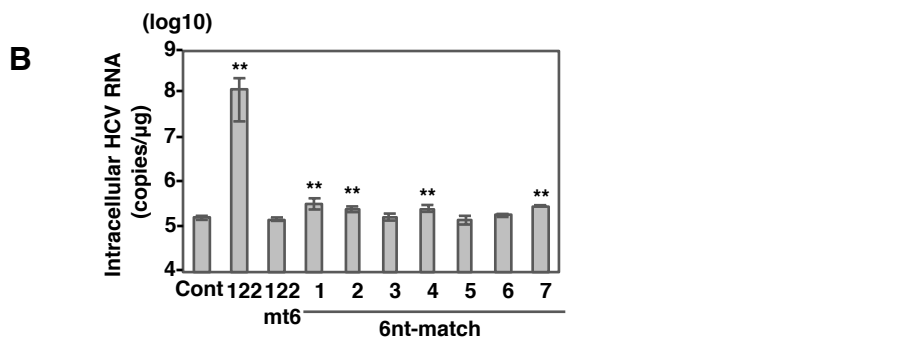

Supplement: S6 Fig — (A) Diagrams of possible interaction between HCV 5’UTR and synthetic miRNAs with 6nt-match. Gray-shaded area indicate possible interaction region. Nucleotides in red are important region for the enhancement by miR-122 shown in Fig 1D. (B) Intracellular HCV-RNA levels of 751- 122KO cells infected with JFH1 in the presence of mimic control, miR-122, miR-122-mt6 and miRNAs with 6nt-match were determined at 72 hpi by qRT-PCR. (PDF) [file ppat.1008308.s006.pdf]

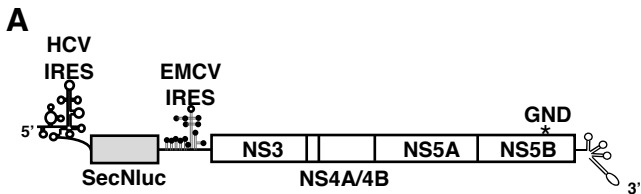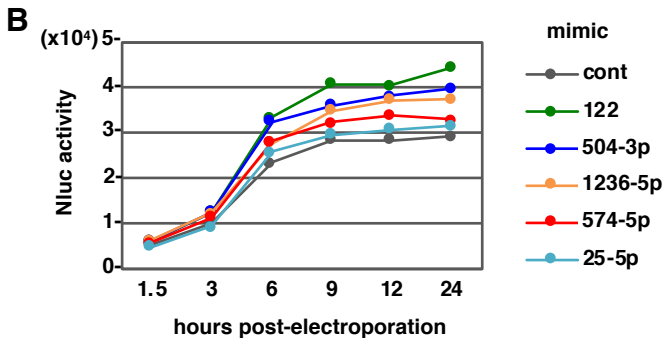

Supplement: S7 Fig — (A) The structure of SGR-GND- JFH1-NlucSec. (B) HCV IRES activity in 751-122KO cells in the presence of mimic control, miR-122, miR-25-5p, miR-504-3p, miR-574-5p and miR-1236-5p was determined by the measurement of NlucSec activity at 1.5, 3, 6, 9, 12 and 24 hours post-electroporation. (PDF) [file ppat.1008308.s007.pdf]

**A**

| HCV-RNA genotype1 | TSI  |
|-------------------|------|
| miR-122-5p        | 0.97 |
| miR-504-3p        |      |
| miR-574-5p        | 0.79 |
| miR-3659          |      |
| miR-1236-5p       | 0.68 |
| miR-4481          | 0.68 |
| miR-4745-5p       | 0.71 |
| miR-4765          |      |
| miR-6880-5p       | 0.56 |

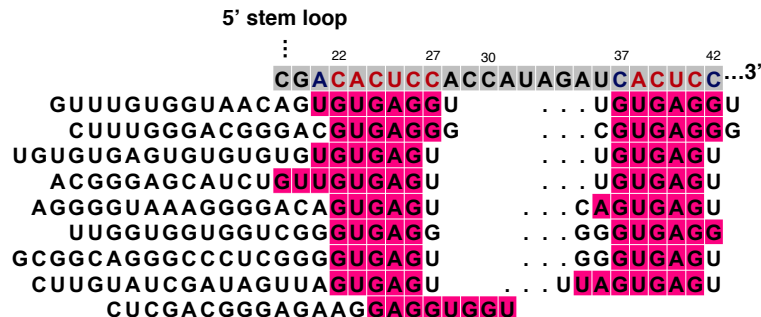

**B**

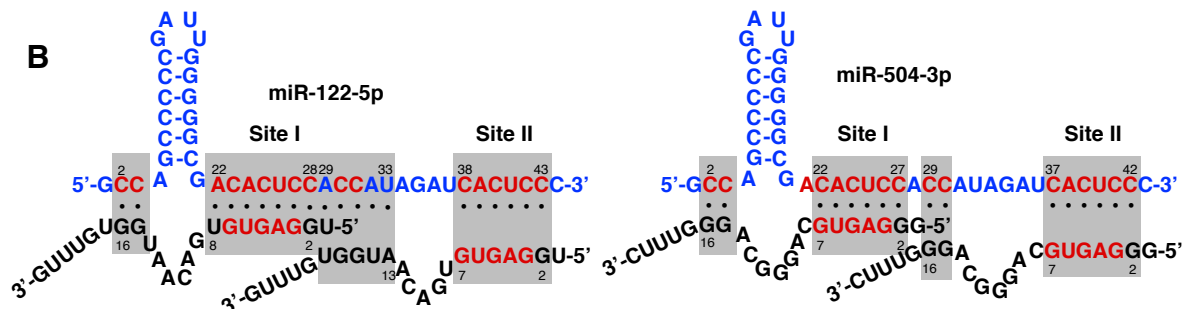

**C**

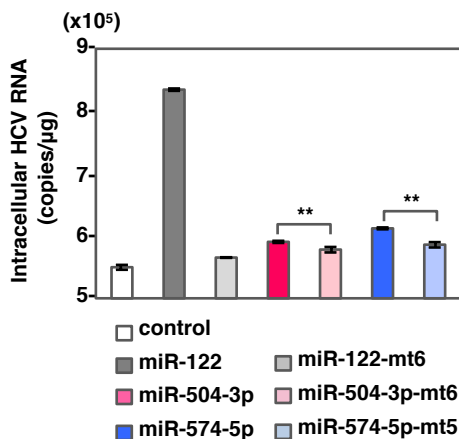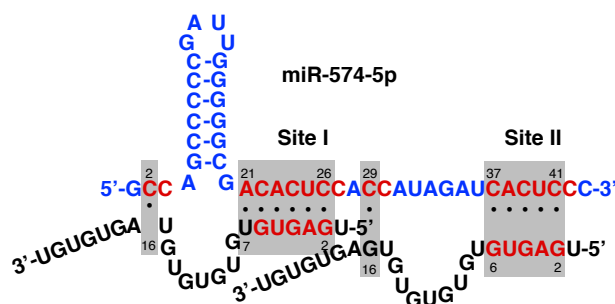

Supplement: S8 Fig — (A) Sequence alignment of interaction sites between nt21-42 of HCV gt1b (Con1 strain) and 5’UTR and candidate miRNAs. Matching nucleotides were shown in red boxes. Nucleotides in site I and II of HCV-RNA were shown in red. (B) Diagrams of possible interaction between Con1 5’UTR and miR-504-3p and miR-574-5p. (C) Intracellular HCV-RNA levels of 751-122KO cells infected with Con1/JFH in the presence of mimic control, miR-122, miR-504-3p and miR-574-5p or their mutant derivatives were determined at 72 hpi by qRT-PCR. The data are representative of three independent experiments. Error bars indicate the standard deviation of the mean and asterisks indicate significant differences (**P < 0.01) versus the results for the control. (PDF) [file ppat.1008308.s008.pdf]

**A**

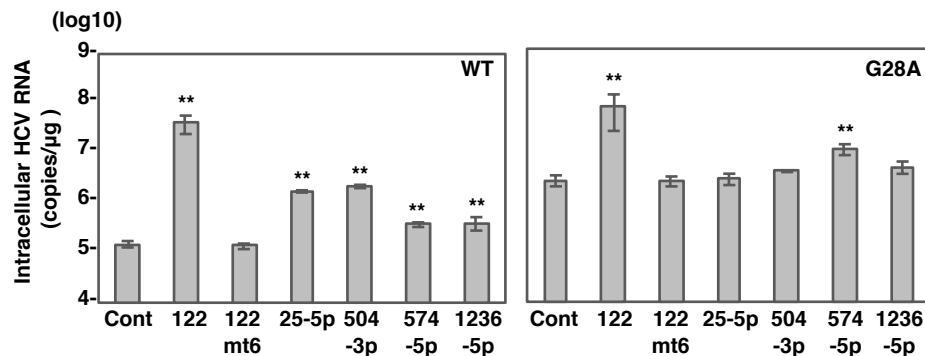

**B**

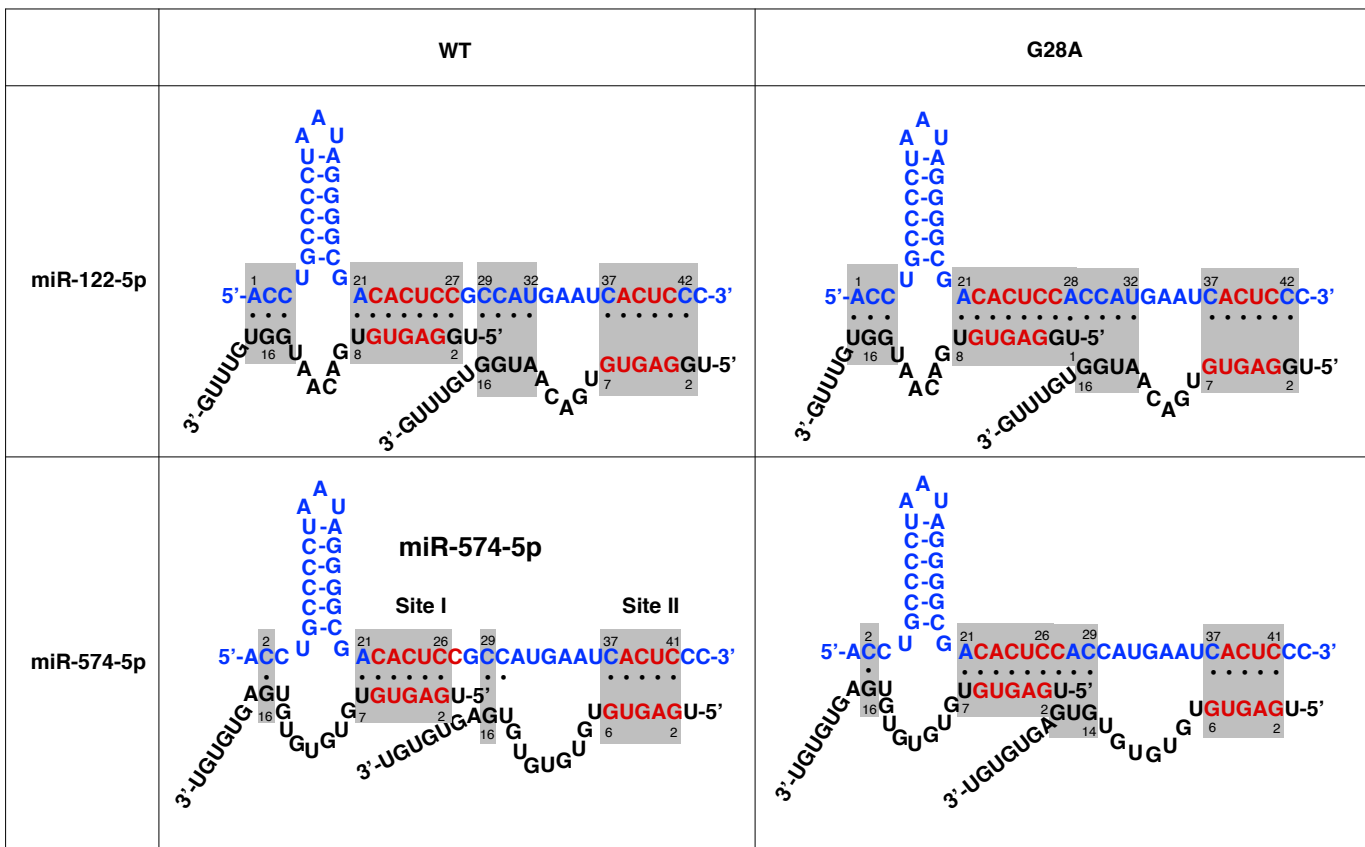

Supplement: S9 Fig — (A) Intracellular HCV-RNA levels of Huh7-122KO cells infected with JFH1 or JFH1-G28A in the presence of mimic control, miR-122, miR-122-mt6, miR-25-5p, miR-504-3p, miR-574-5p and miR-1236-5p were determined at 72 hpi by qRT-PCR. (B) Diagrams of possible interaction between HCV 5’UTR of wild type or G28A mutant and miR-122 or miR-504-5p, respectively. Gray-shaded area indicate possible interaction region. Nucleotides in red are important region for the enhancement by miR-122 shown in Fig 1D. (PDF) [file ppat.1008308.s009.pdf]

| miR-122                                                                            | miR-25-5p                                                                           | miR-504-3p                                                                          | miR-122-GAGUG                                                                       | miR-652-3p                                                                           |
|------------------------------------------------------------------------------------|-------------------------------------------------------------------------------------|-------------------------------------------------------------------------------------|-------------------------------------------------------------------------------------|--------------------------------------------------------------------------------------|
| 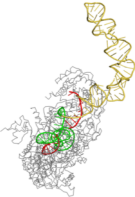   | 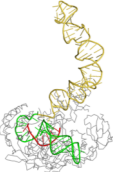   | 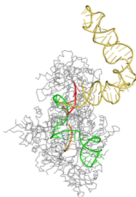   | 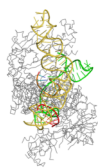   | 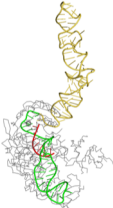   |
| 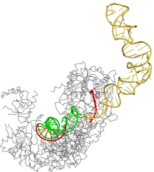   | 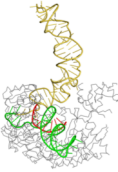   | 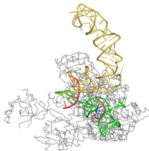   | 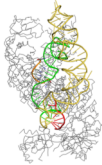   | 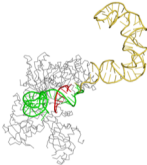   |
| 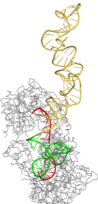  | 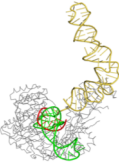   | 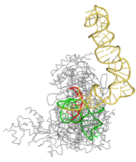   | 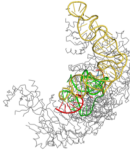   | 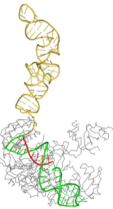   |
| 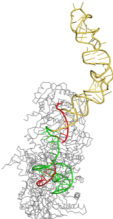  | 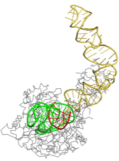  | 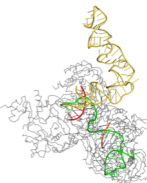  | 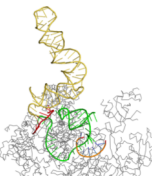  | 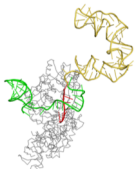  |
| 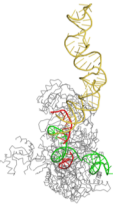 | 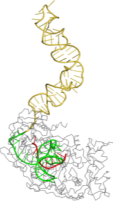 | 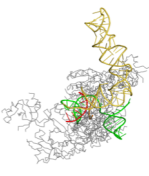 | 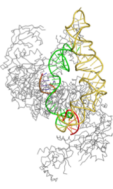 | 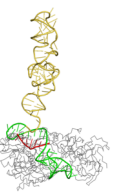 |

Supplement: S10 Fig — miRNA binding regions (nt1-38: green), long arms composed of SLII (nt39-117: orange) of HCV-RNA, miRNAs in double helix at 5’ end (red) and Ago2 (in ribbon representation with gray) were shown. (PDF) [file ppat.1008308.s010.pdf]

**A**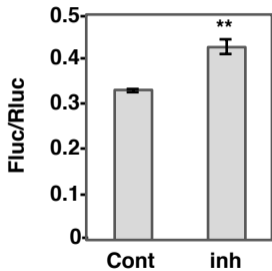**B**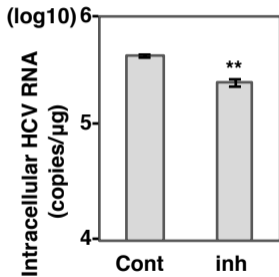

Supplement: S12 Fig — (A) The activities of miR-574-5p in 751-122KO cells. pmirGLO vectors carrying the complementary sequence of miR-574-5p under the luciferase gene was co-transfected with either control miRNA or miR-574-5p inhibitor were transfected into 751-122KO cells. At 24 h post-transfection, the luciferase activity was determined. (B) Intracellular HCV-RNA levels of 751-122KO cells infected with JFH1 in the presence of control miRNA or inhibitor of miR-574-5p were determined at 72 hpi by qRT-PCR. The data are representative of three independent experiments. Error bars indicate the standard deviation of the mean and asterisks indicate significant differences (**P < 0.01) versus the results for the control. (PDF) [file ppat.1008308.s012.pdf]

**A**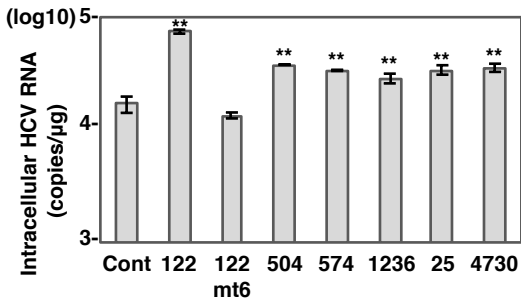**B**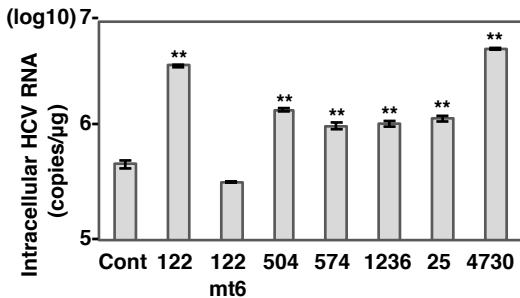

Supplement: S13 Fig — Intracellular HCV-RNA levels of 293T-CLDN1 cells (A) and Hec1B cells (B) infected with JFH1 in the presence of mimic control, miR-122, miR-122-mt6, -504-3p, -574-5p, -1236-5p, -25-5p and -4730 were determined at 72 hpi by qRT-PCR. (PDF) [file ppat.1008308.s013.pdf]
