# Supplementary material for: Various miRNAs compensate the role of miR-122 on HCV replication
Source: PLoS Pathog. 2020 Jun 23;16(6):e1008308. doi: 10.1371/journal.ppat.1008308 (PMC7337399; doi:10.1371/journal.ppat.1008308)
Supplement: S11 Fig — The relative expression of each miRNA was determined by qRT-PCR by using miR-25-3p as an internal control. The values of 2-ΔCT were shown. (PDF) [file ppat.1008308.s011.pdf]

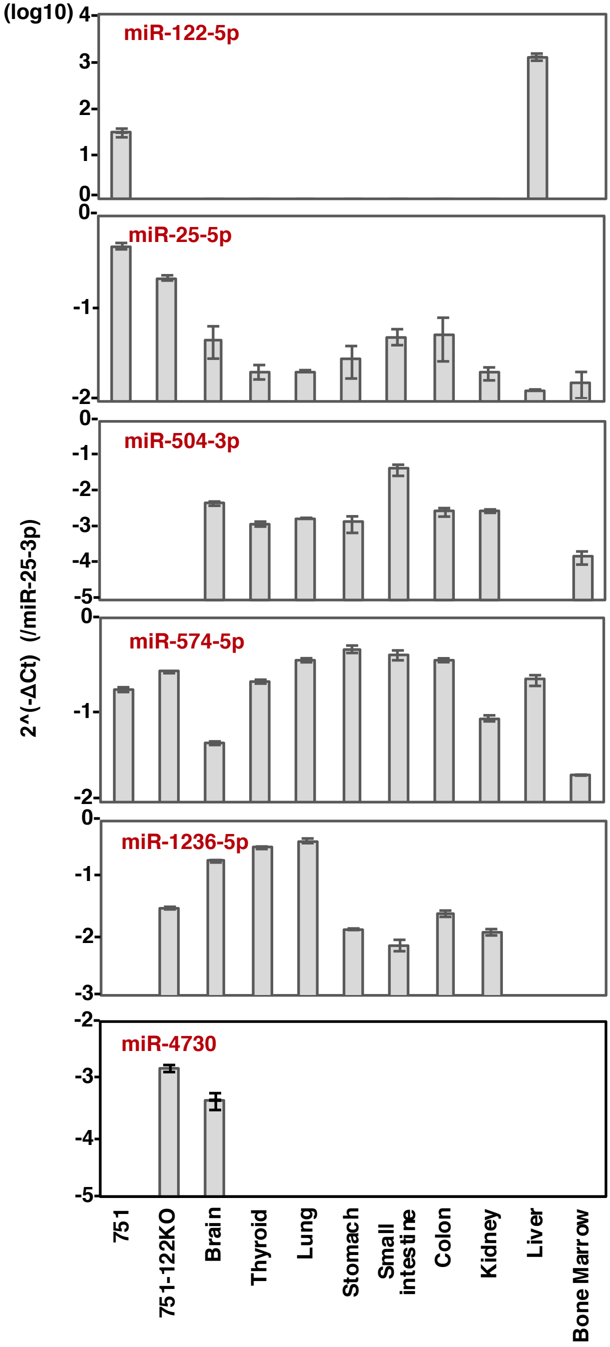
(log10)
miR-122-5p
miR-25-5p
miR-504-3p
miR-574-5p
miR-1236-5p
miR-4730
2^(-ΔCt) (/miR-25-3p)
751
751-122KO
Brain
Thyroid
Lung
Stomach
Small intestine
Colon
Kidney
Liver
Bone Marrow
